# The epidemiology of kuru: monitoring the epidemic from its peak to its end

**DOI:** 10.1098/rstb.2008.0071

**Published:** 2008-11-27

**Authors:** Michael P. Alpers

**Affiliations:** 1 Centre for International Health, ABCRC, Shenton Park Campus, Curtin UniversityGPO Box U1987, Perth, WA 6845, Australia; 2 MRC Prion Unit and Department of Neurodegenerative Disease, UCL Institute of Neurology, The National Hospital for Neurology and NeurosurgeryQueen Square, London WC1N 3BG, UK; 3 Papua New Guinea Institute of Medical ResearchPO Box 60, Goroka, EHP 441, Papua New Guinea

**Keywords:** kuru, epidemiological surveillance, prion diseases, transumption, transmissible spongiform encephalopathies

## Abstract

Kuru is a fatal transmissible spongiform encephalopathy restricted to the Fore people and their neighbours in a remote region of the Eastern Highlands of Papua New Guinea. When first investigated in 1957 it was found to be present in epidemic proportions, with approximately 1000 deaths in the first 5 years, 1957–1961. The changing epidemiological patterns and other significant findings such as the transmissibility of kuru are described in their historical progression. Monitoring the progress of the epidemic has been carried out by epidemiological surveillance in the field for 50 years. From its peak, the number of deaths from kuru declined to 2 in the last 5 years, indicating that the epidemic is approaching its end. The mode of transmission of the prion agent of kuru was the local mortuary practice of transumption. The prohibition of this practice in the 1950s led to the decline in the epidemic, which has been prolonged into the present century by incubation periods that may exceed 50 years. Currently, the epidemiological surveillance is being maintained and further studies on human genetics and the past mortuary practices are being conducted in the kuru-affected region and in communities beyond it.

## 1. Introduction

Kuru is a fatal neurodegenerative disease with a subacute course lasting, on average, 12 months which is found only among the Fore people and their immediate neighbours in the Okapa District of the Eastern Highlands Province of Papua New Guinea (PNG). A description of the general features of kuru may be found in the paper by Collinge *et al.* (2008) in this issue of the *Philosophical Transactions of the Royal Society*. When kuru was first described by medical scientists in 1957, it was a complete mystery. By 1967, it had been essentially solved: kuru was a transmissible spongiform encephalopathy (TSE) transmitted orally through the local mortuary practice of consumption of the dead. A second human spongiform encephalopathy, Creutzfeldt–Jakob disease (CJD), was reported to be transmissible in 1968. The incubation periods of the TSEs were remarkably long compared with that of other infectious diseases. However, the nature of the transmitting agent of kuru and other TSEs remained a mystery until it was shown to be a host-coded protein whose infectivity was determined by its shape, and dubbed a prion in 1982. By then the kuru epidemic had declined dramatically, but the advent of another TSE, bovine spongiform encephalopathy (BSE), and its oral transmission to humans to cause variant CJD (vCJD), led to a renewed global interest in kuru in the late 1990s.

If epidemiology is to have any explanatory power it must be multidisciplinary, and kuru exemplifies this in a striking way. I shall give here a brief account from a personal perspective of how these epidemiological and explanatory events unfolded for kuru.

## 2. Geography and history

The geographical location of the kuru region within the eastern central highlands of PNG is shown in figure 1. Its position within PNG is more broadly shown in the first figure of Collinge *et al.* (2008), where the Fore linguistic group (which has had over 80% of the recorded cases of kuru) is divided into South Fore and North Fore; the remaining nine linguistic groups are affected by kuru in the part of their population adjacent to the Fore. In the more extended part of the highlands shown in figure 1, there are many linguistic groups; in PNG as a whole, there are approximately 850 (a seventh of the world's languages in a population of 5 million people). The kuru region is approximately 65 by 40 km in size and in the late 1950s contained approximately 40 000 people.

PNG is a remarkable country for many reasons, not only linguistic, and New Guinea is a fabulous island famed in Asia for its birds of paradise for millennia (though not known to Europeans until the sixteenth century). A million people lived in the highlands of New Guinea in 1930, whose existence was unknown to the rest of the world. The international boundary across the middle of the island is a colonial artefact. The Australian-administered side of the island (now the independent nation of PNG) began to infiltrate its control into the highlands in 1930. However, the Okapa area where kuru occurs was not opened up to administrative control, when the lives of its people changed forever, until the 1950s (see Alpers 2008).

Research on kuru began in 1957 (Gajdusek & Zigas 1957). One of the first achievements was to determine the geographical boundaries of kuru, which was carried out by Gajdusek *et al*. (1961). The second figure in Collinge *et al.* (2008) shows the location of all the villages with a history of kuru; of the 172 villages, 155 have had a case reported since 1957 and are included in the kuru database. The geographical definition of these villages as the kuru region is described by an enclosed area in figure 1. The boundary is very sharp in the southeast since all 65 Fore villages have had many cases of kuru, but the Anga, living across the Lamari River, have had none. In the north and west, the boundary fades out among the Auyana, Usurufa, Kamano, Kanite, Yate, Yagaria, Keiagana and Gimi linguistic groups. There have been only three cases in the Awa. Considering the steep, dissected nature of the terrain and the fact that each village consists of hamlets scattered over the landscape, establishing the boundaries of the kuru region was a formidable task and to complete it so quickly and efficiently an extraordinary achievement.

The colleagues who worked with Gajdusek and Zigas in 1957 are acknowledged in the second paper on kuru (Zigas & Gajdusek 1957) and the sequence of research workers who followed is given in the Introduction to these proceedings (Collinge & Alpers 2008). Indeed, the reminiscences and reflections of many of them are included in the proceedings. The contributions of others to our knowledge about kuru are described by Alpers (2008).

From oral history, the epidemic of kuru began early in the twentieth century in Uwami village on the edge of the kuru region, spread to Awande and down the valley to Kasokana, and from there north into the North Fore and beyond and, a little later, but most dramatically, to the South Fore and Gimi. The historical spread of kuru has been described by Glasse (1962) and Mathews (1965).

## 3. Clinical and pathological definition

The clinical features define kuru as a progressive cerebellar disease (Simpson *et al.* 1959; Zigas & Gajdusek 1959; Alpers 1964*a*,*b*; Hornabrook 1968). It is much more than that with a wide variety of transient motor signs found in patients followed carefully from onset to death. The progression of the cerebellar disease is clearly divided into three stages: ambulant, sedentary and recumbent, which often has a prolonged terminal state. The disease begins with pain (headache and joint pain); because this occurs for a variable period before the onset of cerebellar ataxia, it has been regarded, for clinical and epidemiological purposes, as a prodrome. Because such symptoms are common in the absence of kuru, particularly more recently with the increase in malaria in the highlands, and because kuru is the disease uppermost in people's minds in the region, false reports of kuru are common and require careful attention in epidemiological surveillance (they are recorded within the ‘redherring’ file of the database as recovery, rejected, reassigned or relinquished). No true recovery has ever been documented in a patient showing established signs of cerebellar ataxia; however, some patients have a long period of fluctuating illness at the beginning of their clinical course before settling into progressive, ultimately fatal, cerebellar disease.

The fact that other clinical signs of disease, involving the brain stem, mid-brain, hypothalamus and cerebral cortex, are found during the course of kuru is not surprising considering the extent of the pathological change at post-mortem. The neuropathology was initially described by Fowler & Robertson (1959) and Klatzo *et al.* (1959). The cardinal features are neuronal loss and degeneration, often with vacuolation; astrocytic hypertrophy and proliferation; spongiform change (from vacuoles in neuronal processes); the absence of inflammation; and, in some cases, PAS-positive (amyloid) plaques. The histopathological changes were found throughout the brain and spinal cord but were most pronounced in the cerebellum. These features were not all so confidently delineated in 1959, but when William J. Hadlow, a veterinary neuropathologist, saw an exhibition of kuru in that year and examined the neuropathology (Hadlow 2008), he was immediately struck by the similarity to scrapie. Scrapie is a spongiform encephalopathy of sheep and the first such disease shown to be transmissible, with very long incubation periods even when transmitted to sheep. The implications for kuru were clear: the need to undertake transmission studies in higher primates (Hadlow 1959). Transmission studies in chimpanzees were therefore planned and slowly implemented, as funds, animal facilities and animals were acquired. The aim was to provide impeccable inocula taken soon after death (see Alpers 2008), to inoculate a host as close as possible to humans (the chimpanzee) and to follow the inoculated animals rigorously for 10 years before declaring a negative outcome.

## 4. Distribution of kuru

The geographical distribution has been described. The geographical distribution of cases mapped by village and plotted at different time points is shown in Alpers & Kuru Surveillance Team (2005). The sex and age distribution in 1957–1959 proved to be remarkable, with 60 per cent of cases in adult females, only 2 per cent in adult males and the remainder in children and adolescents of both sexes (Alpers & Gajdusek 1965). The youngest case was aged 4 years at onset and the oldest over 60 years.

## 5. Changing epidemiological patterns

In 1964, I reviewed all the recorded cases of kuru in the clinical files held in Dr Carleton Gajdusek's laboratory at the National Institutes of Health (NIH) in Bethesda, Maryland and compared the first 3 years of the epidemic (1957–1959) with the most recent 3 years (1961–1963). When the figures were compiled by age and sex, a remarkable finding was revealed, shown in figure 2 by two superimposed histograms (such figures at that time grew under your hand as they were created and the revelation of this one generated considerable excitement). Kuru in the later period had almost disappeared in the youngest age group in both males and females. The bimodality of the distribution in females in the earlier period is clearly evident; this was not found in males and by the later period had essentially gone. The other point to note is the appearance of kuru in older males in 1961–1963. Further changing epidemiological patterns in time and space, during the first 7 years of kuru surveillance, are described by Alpers (1965) and Alpers & Gajdusek (1965).

## 6. Transmission experiments

Meanwhile, the transmission experiments in chimpanzees were underway. The principal aims of the experiment have been outlined above and further details are provided in Alpers (2008). In following the inoculated animals as planned, by clinical examination and cinema documentation, I became for a while probably the busiest chimpanzee neurologist in the Washington area. As it turned out, we did not have to wait for 10 years to establish a negative outcome: the chimpanzees began to come down after approximately 2 years' incubation with behavioural changes that soon developed into unequivocal cerebellar ataxia. The positive outcome of transmission of kuru to the chimpanzee had been achieved (Gajdusek *et al.* 1966) and was confirmed pathologically (Beck *et al.* 1966).

In Alpers (2008), I honoured some of my colleagues for their contributions to our understanding of kuru. Here I wish to acknowledge other contributions: that of the patients who agreed to have an autopsy performed on their body soon after their death; and the chimpanzees who suffered for the cause of science. In the circumstances, these relationships involved much more than a standard professional duty of care. In the paper reporting the transmission (Gajdusek *et al.* 1966), the donors and recipients of infective brain tissue were named, as a matter of honour. The first autopsy I performed was on a boy whose first name was Eiro; his tissue was inoculated into Georgette, who, though not the first to be inoculated, was the first to come down with kuru. The second chimpanzee to come down was Daisey, who had been the first to be inoculated; she was inoculated with tissue from Kigea, a girl whom I knew very well. I followed my patients in their home setting from onset to death; in Kigea's case, there was a long period of 6 weeks when she lingered in her terminal moribund state subsisting on sips of sugar water, unable to move her body but still aware of her surroundings, and still capable of making eye contact and a flicker of recognition whenever I came to visit her. I am proud to have known her and her family.

## 7. Human behaviour

There were many remarkable things about the people of the kuru-affected region; one was the means of disposal of the dead, which was normally by consumption of the body by the dead person's kin. This practice was investigated by all of us who worked in the area but particularly by the two anthropologists Robert Glasse and Shirley Lindenbaum, who worked there as a team (Glasse 1967; Lindenbaum 1979).

Owing to the derogatory connotations of the word ‘cannibalism’, which are only softened a little by the more technical ‘endocannibalism’, I have adopted the term ‘transumption’ to describe the mode of disposal of the dead practised by the Fore and their neighbours: as defined in Alpers (2007) this is ‘the mortuary practice of consumption of the dead and incorporation of the body of the dead person into the bodies of living relatives, thus helping to free the spirit of the dead’. There could also be considerable benefits to the living from this practice, but it was fraught with many dangers (Whitfield *et al.* 2008).

The whole body was eaten by the female relatives and their children of both sexes. Adult males, which included boys above the age of approximately 7 years who were living in the men's house, rarely partook of the body and never ate the brain or other internal organs.

## 8. Human genetics

The initial most favoured explanation for kuru was a genetic one, though every possible cause was considered by the early investigators (Gajdusek 1963). Another mechanism that was considered a strong possibility was autoimmune disease as a consequence of eating human brain tissue. A genetic hypothesis for kuru was proposed (Bennett *et al.* 1959). At the same time, studies on the human genetics of the linguistic groups of the kuru region and other parts of PNG were carried out (Simmons *et al.* 1961).

## 9. Epidemiological explication, proof, prediction and intervention

All the information was to hand by 1966 to solve the puzzle of kuru and resolve at least some of its mystery. Clearly, one or more of the many changes that had occurred in the kuru region (Alpers 1965) could be the explanation for the dramatic decline in the incidence in young children. There was a reluctance to attribute this change to the decline in ‘cannibalism’ (Alpers 1965). However, the proven involvement of a transmissible agent in kuru meant that transumption could provide the mode of transmission of the agent rather than being the primary cause itself. The details of the mortuary practices readily explained the sex and age distribution of kuru. The practice had been proscribed by the Australian administration as one of their first acts of ‘control’ and public feasting had ceased by the mid-1950s and the practice abandoned by the early 1960s. This would create a cohort effect with children born since this time growing up free of the disease. Current cases could be explained by the long incubation period characteristic of TSEs. Finally, in 1967, it all came together (Alpers 1968).

A corollary of this explanatory argument was that kuru was not transmitted vertically from mother to child since many mothers had been pregnant, given birth or breastfed their babies while they had kuru and no such children were coming down with the disease. This argument is a good illustration of how logical proof can be part of epidemiology as well as statistical proof.

With the complete break in transmission, a progressive decline in the epidemic was predicted, its length dependent on the incubation periods of the remaining infected individuals. The cohort effect would lead to a steadily progressive increase in the age of the youngest case. Though the formal genetic hypothesis to explain kuru was no longer tenable, genetics could still play an important role in the pathogenesis of disease in the host exposed to the infective kuru agent.

Every epidemiological programme should include consideration of an intervention to reduce the burden of the disease being studied. Kuru is unusual, as in so many other ways, in that the intervention came first and provided an important clue that enabled, with other evidence, the aetiological puzzle to be solved; this was then able to explain why the unwitting intervention had worked.

Mathews *et al.* (1968) came to similar conclusions from the multidisciplinary data available. When the infectious nature of kuru, which had always been one of the available hypotheses, had become a fact, the puzzle could be pieced together since it is so much easier to develop a convincing explanatory model from facts than from hypotheses.

## 10. Subsequent events

Epidemiological surveillance in the field continued (Alpers 1979, 1987; Alpers & Kuru Surveillance Team 2005) and still continues (Collinge *et al.* 2008; Pako 2008); the methodology has been described by Alpers & Kuru Surveillance Team (2005). The kuru database has been maintained. The fruits of this labour may be seen in figure 3, which describes the curve of the kuru epidemic from its peak in the period 1957–1961 to its end, or almost its end: though we do not know how many more cases of kuru there will be, we can expect only a few more at the most.

After kuru had been found to be transmissible and its mode of transmission worked out, the origin of kuru still remained an unsolved question. The solution proposed by Alpers & Rail (1971) is now generally accepted: that kuru arose in a single individual from a spontaneous change that created a pathogenic, infectious agent in the brain, in the same way as sporadic Creutzfeldt–Jakob disease arises (the concept as then formulated was not dependent on the nature of the agent, and would have fitted an endogenous virus or a host-coded protein-only agent). Such a chance event, though rare, leads to sporadic CJD in approximately one person in every million each year in all human populations. Kuru may have begun as an ataxic variant of CJD or may have become so through oral transmission. The practice of transumption is in itself not risky and many communities outside the kuru-affected region practised it with impunity. However, add to a community practising transumption a person dying of a TSE: the intraspecies recycling of the infectious agent through transumption (when every dead person was completely eaten by kinfolk) amplifies both the agent and the disease in the community, and creates an epidemic. Genetic evidence suggests that this may have happened not infrequently in the remote human past (Mead *et al.* 2003), and may happen too with other species.

Studies on human behaviour and exploring the details of transumption are being continued (Whitfield 2008; Whitfield *et al.* 2008). This may provide a basis for understanding the historical spread of kuru within the region. The study of human genetics in relation to kuru expanded initially (Gajdusek & Alpers 1972) but then paused. The unconventional kuru agent, initially conceived of as a slow virus, became, with the agents of other TSEs, a prion (Prusiner 1982), a protein-only infectious agent. The prion is host coded and the genetics of the human prion protein gene has proven to be of particular interest in kuru (Mead *et al.* 2003), surpassing all predictions.

The incubation period in males has been documented in recent cases to be 50 years or more (Collinge *et al.* 2008), since males were exposed only in the first 7 years of their lives while they were with their mothers. Because we now know how long the incubation period may be, we believe that the small number of adult male cases in the past all arose from long incubation periods after childhood exposure. This may explain the first cases in older males seen in the later period of 1961–1963 in figure 2: this age group had not had cases before because the epidemic had been going for only approximately 40 years, or even less in parts of the South Fore, so that older males in the earlier period of 1957–1959 would have left the care of their mothers and the risk of exposure through transumption before the probability of exposure to kuru in their communities became high. An explanation for the bimodality seen in females in 1957–1959, based on transumption practices, is given in Alpers & Hörnlimann (2007). As well as the documented long incubation periods in some cases, the incubation period may often have been as short as 2 years, even from oral transmission; the best estimate for the mean incubation period is approximately 12 years.

We expect the epidemic to be over soon, based on the pattern of decline in communities where kuru has indeed come to an end. The last death was in 2005 and there is no known current case. In order to be able to state this with certainty, we have to continue rigorous epidemiological surveillance. Our exit strategy is to continue surveillance until there is a period of 5 years with no kuru after the death of the last case.

The other significant events that occurred in the field of the transmissible spongiform encephalopathies, the epidemic of BSE (through the intraspecies recycling of bovine brain in meat-and-bone meal fed to calves) and the transmission of BSE to humans causing vCJD, which have led to renewed interest in kuru, are described by Collinge *et al.* (2008). Kuru may be essentially gone, but it is still an important model and its influence is likely to last long into the future.

## Figures and Tables

**Figure 1:**
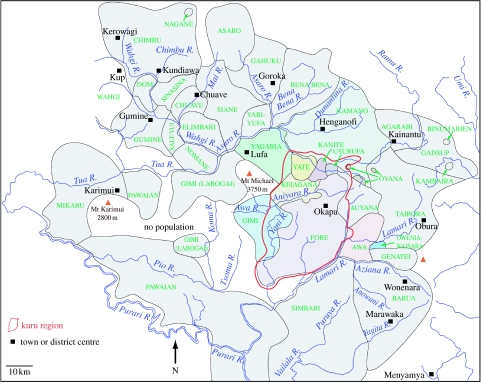
Map showing the linguistic groups of the eastern central highlands of PNG, major rivers and mountains, towns and the kuru-affected region. From Alpers (2007), where the history of earlier versions of the map can be found. Reproduced with permission of the Papua New Guinea Medical Journal.

**Figure 2:**
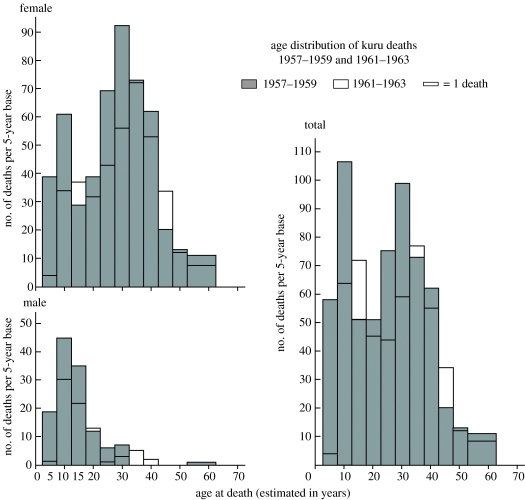
Histogram comparing the sex and age distribution of all deaths from kuru in 1957–1959 with that in 1961–1963. Adapted from Alpers (1965).

**Figure 3:**
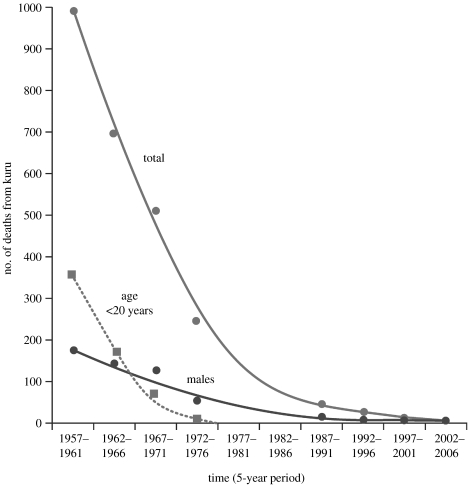
Graph showing the decline of the kuru epidemic from its peak in 1957–1961 to its imminent end. The data from the years 1977–1986 are not yet available.
